# Subjective outcome related to donor site morbidity after sural nerve graft harvesting: a survey in 41 patients

**DOI:** 10.1186/1471-2482-13-39

**Published:** 2013-09-24

**Authors:** Alexander Hallgren, Anders Björkman, Anette Chemnitz, Lars B Dahlin

**Affiliations:** 1 Department of Clinical Sciences Malmö, Hand Surgery, Lund University, Malmö, Sweden

**Keywords:** Sural nerve, Nerve reconstruction, Nerve injury, Cold intolerance, Allodynia, Pain

## Abstract

**Background:**

The sural nerve is the most commonly used nerve for grafting severe nerve defects. Our aim was to evaluate subjective outcome in the lower leg after harvesting the sural nerve for grafting nerve defects.

**Methods:**

Forty-six patients were asked to fill in a questionnaire to describe symptoms from leg or foot, where the sural nerve has been harvested to reconstruct an injured major nerve trunk. The questionnaire, previously used in patients going through a nerve biopsy, consists of questions about loss of sensation, pain, cold intolerance, allodynia and present problems from the foot. The survey also contained questions (visual analogue scales; VAS) about disability from the reconstructed nerve trunk.

**Results:**

Forty-one out of 46 patients replied [35 males/6 females; age at reconstruction 23.0 years (10–72); median (min-max), reconstruction done 12 (1.2-39) years ago]. In most patients [37/41 cases (90%)], the sural nerve graft was used to reconstruct an injured nerve trunk in the upper extremity, mainly the median nerve [19/41 (46%)].

In 38/41 patients, loss of sensation, to a variable extent, in the skin area innervated by the sural nerve was noted. These problems persisted at follow up, but 19/41 noted that this area of sensory deficit had decreased over time. Few patients had pain and less than 1/3 had cold intolerance. Allodynia was present in half of the patients, but the majority of them considered that they had no or only slight problems from their foot. None of the patients in the study required painkillers. Eighty eight per cent would accept an additional sural nerve graft procedure if another nerve reconstruction procedure is necessary in the future.

**Conclusions:**

Harvesting of the sural nerve for reconstruction nerve injuries results in mild residual symptoms similar to those seen after a nerve biopsy; although nerve biopsy patients are less prone to undergo an additional biopsy.

## Background

The sural nerve is located on the back of the lower leg. It is formed by joining the medial sural cutaneous nerve with the peroneal branch of the lateral sural cutaneous nerve where after it runs down along the leg. It pierces the fascia in the middle of the leg and is located superficially in the fat together with the lesser saphenous vein behind the lateral malleolus. The nerve innervates the skin mainly around the heel, but in some individuals it innervates also the lateral side of the foot, including the little toe [1].

The sural nerve is used in many ways in today’s medicine; it can be used in nerve biopsies to assist in diagnosing polyneuropathies of unclear origin and to detect efficiency of pharmacological substances to treat neuropathy [2,3]. It is important that the indications for a sural nerve biopsy are accurate [4] since pain and discomfort may develop following the biopsy. In addition, the sural nerve is the most common autologous donor nerve, in nerve grafting, to reconstruct severe nerve defects in both adults and children. Although the sural nerve is extensively used as a donor nerve, a limited number of studies have investigated the sequelae following harvesting of the sural nerve [5-9], particularly as related to the symptoms seen in subjects and patients that have undergone a sural nerve biopsy.

To be able to inform patients about possible residual symptoms and subjective outcomes when the sural nerve is used as a nerve graft or for a biopsy, knowledge about pain, sensory deficit, allodynia and other problems following harvesting of the sural nerve have to be improved. Based on such knowledge, patients can be offered more accurate and detailed information about possible sequelae following the procedure, which should be put into perspective of the expected result of the nerve reconstruction. Therefore, it is of interest to analyse to which extent the symptoms occur when the sural nerve is used as a nerve graft and also to compare this to the symptoms that are seen after a sural nerve biopsy. Thus, our aim was to evaluate subjective outcome in the lower leg after harvesting the sural nerve for nerve grafting.

## Methods

### Patient material

Patients, operated on with nerve reconstruction at our department between 1973 and 2010 with one or two sural nerves (harvest of the whole length of the sural nerve at reconstruction) used as nerve grafts, were identified by the hospital´s patient registration system. Exclusion criteria were reconstruction of the sciatic or tibial nerves on the same side as the sural nerve graft was harvested and follow up less than 14 months. Forty-six patients filled the criteria and were asked to fill out a questionnaire [4,10] to describe symptoms from the leg or the foot where the nerve graft was harvested. The different parameters investigated included gender, age at reconstruction, age at follow up, time since injury, type of injured nerve, cause of injury, type of incision on leg and the patient´s occupation. The Central Ethical Review Board in Lund (i.e. regional ethics committee) judged the study and found it sound. A formal approval was not necessary, since such a study is not included in the applicable law (Research in humans; law 2003:460). Thus, there were no ethical problem involved (2011/607). Therefore, no formal informed consent was needed from each patient, which was not considered to affect the results.

### Questionnaire

In the autumn of 2011 a questionnaire, used previously, but slightly modified to better fit the present patients, to evaluate residual subjective symptoms following sural nerve biopsies in subjects with or without diabetes [4,10], were sent to the 46 identified patients (Additional file 1). It consists of 14 different questions where the patients have to subjectively evaluate their post-operative symptoms from the lower leg. It includes loss of sensation, pain in the operated area (and if so if the patient had to take painkillers to cope with the pain), cold intolerance, numbness and tingling sensation.

A scoring system, based on the patient´s subjective perceptions and symptoms from the questionnaire, was created to compare any symptoms and discomfort with the overall general outcome of the reconstructed nerve. The scoring system was designed as:

I.) Do you have loss of sensation in the operated foot compared to the other foot? [no = 0; yes = 1].

II.) Do you feel pain in the foot/lower leg? [no = 0; day time = 1; night time = 2].

III.) Do you have problem with cold intolerance in the operated foot/lower leg? [no = 0; rarely = 1; sometimes = 2; frequently = 3].

IV.) Have you experienced problems with increased skin sensation when the skin is touched? [no = 0; rarely = 1; sometimes = 2; frequently = 3].

V.) Do you experience discomfort or tingling along the outside of the foot? [no = 0, impacts against surgical site = 1; during walking = 2; at rest = 3].

VI.) How would you describe your problems at the moment? [none = 0; mild = 1; affecting daily living = 2; severe = 3; disturbed sleep = 4].

The survey also contained three different visual analogue scales (VAS) focusing on the outcome on the patients by the injured and reconstructed nerve. The patients were asked to rate a) at present how much does your nerve injury, i.e. the reconstructed nerve, affect you? b) what impact has your nerve injury had on leisure activities and c) how does your injury affect your work or school attendance?

For comparison, the results from a previous study, using the same questionnaire, describing the postoperative complaints after a whole, not a fascicular, sural nerve biopsy had been taken in 21 subjects without diabetes [4].

### Statistics

Values are presented as median (min – max) or numbers (%). Mann Whitney *U*-test was used to test for any significant difference between patients with an age ≤ or > 20 years of age at injury and Kruskal-Wallis to detect any differences between the various injuries leading up to the nerve grafting procedures. Correlations were done with the Spearman correlation test. A p-value of < 0.05 was accepted as significant. Analyses were done with using StatView for windows (SAS Institute Inc, Cary, NC, USA, version 5.0.1) and IBM SPSS Statistics (Statistical Package for the Social Sciences, SPSS Inc., Chicago, Il, USA) version 20 for Mac.

## Results

Patients’ characteristics are shown in Table 1. Forty-one out of 46 patients responded to the questionnaire [35 males/6 females; median age at reconstruction was 23 years (min-max 10–72)]. The nerve reconstructions were done at a median of 12 years (min-max 1.2-39 years) ago. In 22/41 (54%) of the patients multiple incisions were used and in 6/41 (15%) of the cases a single longitudinal incision was the surgical technique of choice when harvesting the sural nerve. In 13/41 (32%) of the cases the surgical notes did not say which type of incision the surgeon used. There were no reports of postoperative complications, such as infection or deep vein thrombosis, in any of the patients included in this study, where the whole length of the sural nerve was harvested.

**Table 1:** Patient characteristics

		
Gender [male/female]:		35/6 (85/15%)
Age at reconstruction [years]:		23.0 (10–72)
Time since reconstruction [months]:		144 (14–468)
Reconstructed nerve:	Nerves in lower extremity	4/41 patients
	Nerves in upper extremity	40/41 patients
	Brachial plexus	3/41 patients
Occupation:	Student	9/41 (22%)
	Non-manual labour	4/41 (10%)
	Manual labour	15/41 (37%)
	Retired	1/41 (2%)
	Unknown	12/41 (29%)

Values are numbers (%) or median (min-max).

### Residual symptoms from the foot and leg

The residual symptoms following harvesting of the sural nerve did not depend on type of injury for which the nerve was harvested (p = 0.58). In most patients [37/41 cases (90%)], the sural nerve graft was used to reconstruct one or several nerve trunks in the upper extremity; mainly the median nerve [(19/41 (46%); Table 1]. Discomfort from the leg was noted in 26/41 (63%) of the patients directly following surgery (Table 2). 38/41 (93%) had loss of sensation, to a variable extent, in the skin areas innervated by the sural nerve, which persisted at follow up. However, 19/41 (46%) patients noted that this area had decreased over time. Immediately following the operation, 18/41 (44%) experienced pain in the foot, while 21 (51%) did not (no reply from two patients). The pain did not, however, presently require more potent painkillers in any of the cases. Instead, it was consistently graded as mild by the patients and only 3/41 (5%) had pain during night-time.

**Table 2:** Questionnaire

	**Present patient (n = 41)**	**Healthy patients with sural nerve biopsy (Dahlin et al. 1997) [4]: (n=21)**
1. Did you have any discomfort in the foot directly after the operation?		
Yes	15 (37%)	2 (10%)
No	24 (59%)	19 (90%)
No reply	2 (5%)	
2. Did you experience any loss of sensation in the operated area after the operation?		
Yes	38 (93%)	19 (90%)
No	3 (7%)	2 (10%)
3. Did you experience pain in the operated area after the operation?		
Yes	21 (51%)	7 (33%)
No	18 (44%)	14 (67%)
No reply	2 (5%)	
4. Do you have loss of sensation in the operated foot compared with the other foot?		
Yes	35 (85%)	19 (90%)
No	6 (15%)	2 (10%)
5. Mark the area of sensory deficit in the figure.		
	See Figure 1 for details. 5 (12%) did not draw at all.	
6. (a) Has the area with loss of sensation decreased compared with the time directly following surgery?		
Yes	19 (46%)	8 (38%)
No	22 (54%)	13 (62%)
(b) If yes, how much (%)		
0-25	3 (7%)	2 (10%)
26-50	6 (15%)	1 (5%)
51-75	7 (17%)	3 (14%)
76-100	3 (7%)	2 (10%)
7. (a) Do you feel pain in the foot/lower leg?		
Yes	8 (20%)	1 (5%)
No	33 (80%)	20 (95%)
(b) When?		
Day time	5 (12%)	0 (0%)
Night time	2 (5%)	0 (0%)
Day time and Night time	1 (2%)	1 (5%)
8. (a) Do you have problems with cold intolerance in the operated foot/lower leg?		
Yes	12 (29%)	1 (5%)
No	29 (71%)	20 (95%)
(b) If yes, how often?		
Frequently	3 (7%)	0 (0%)
Sometimes	6 (15%)	0 (0%)
Rarely	3 (7%)	1 (5%)
9. (a) Have you experienced problems with increased skin sensation when the skin is touched?		
Yes	21 (51%)	7 (33%)
No	20 (49%)	14 (67%)
(b) If yes, how often?		
Frequently	7 (17%)	0 (0%)
Sometimes	5 (12%)	4 (19%)
Rarely	9 (22%)	3 (14%)
10.(a) Do you experience discomfort or tingling along the outside of the foot?		
Yes	22 (54%)	10 (48%)
No	19 (46%)	11 (52%)
(b) If so, when do these symptoms occur?		
At rest	6 (15%)	3 (14%)
During walking	4 (10%)	1 (5%)
Impact against surgical site	15 (37%)	6 (29%)
11. How would you describe your problems at the moment?		
Disturbed sleep	0 (0%)	0 (0%)
Powerful	2 (5%)	1 (5%)
Affecting daily living	5 (12%)	1 (5%)
Mild	19 (46%)	11 (52%)
None	16 (39%)	8 (38%)
12. Do you have to take painkillers often?		
Yes	0 (0%)	0 (0%)
No	37 (100%)	21 (100%)
13. (a) Do you have any disease that can affect the nervous system, for example; diabetes, vitamin deficiency or thyroid disease.		
Yes	3 (7%)	-
No	38 (93%)	-
(b) If yes, which?		
	Diabetes mellitus	-
	Thyreoiditis	
14. A theoretical question: would you be positive to have your other sural nerve harvested if you had to undergo another nerve reconstructive surgery?		
Yes	36 (88%)	7 (33%)
No	5 (12%)	14 (67%)
Right now how much does your nerve injury affect you? (VAS)	25 (0–100)	-
[0 = no impact at all; 100 = severe impact]		
What impact has your nerve injury had on leisure activities? (VAS)	8.5 (0–100)	-
[0 = no impact at all; 100 = severe impact]		
How does your injury affect your work or school attendance? (VAS)	11.5 (0–100)	-
[0 = no impact at all; 100 = severe impact]		

**Figure 1:**
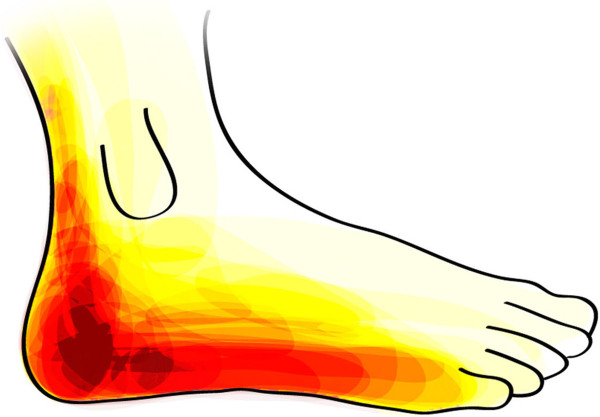
**A combined illustration, i.e. a topographic map, of all the patients’ drawings of the subjective sensory loss.** The more intense the red colour of certain areas was, the more patients have experienced symptoms from this area. In 5/41 (12%) cases, there was no drawing.

The patients were asked to mark the area of the skin with sensory loss after harvesting (question number five). The image (i.e. a topographic map) displayed in Figure 1 shows a combined illustration of all the patients’ drawings. The more intense red colour of certain areas, the more patients have experienced symptoms from this area. The results indicate that 93% of the patients noticed impaired sensation around the heel. In 5/41 (12%) cases, there were no drawings at all in the survey.

Less than one third of the patients had symptoms, such as cold intolerance, and of those that had; only three classified them as frequent. Instead, the most common grade was “sometimes” [6/41 (15%); Table 2]. Allodynia was present in half of the patients 21/41 (51%), and of those affected the most common occasion was “rare” [9/21 (43%)]. A similar number of patients [i.e. 22/41 (54%)] experienced discomfort or tingling sensations along the lateral side of the foot. However, they only perceived such symptoms when the area of previous incision was percussed [15/41 (37%)]; few patients [6/41 (15%)] had tingling sensations in the foot at rest.

On the question on how the symptoms from the sural nerve harvest affected the patients at the moment, 2/41 (5%) classified it as powerful; 5/41 (12%) felt that it affected their daily activities; and the majority 35/41 (85%) responded that it was mild or none at all. No patients had sleeping problems due to symptoms. If necessary in the future 36/41 (88%) patients were positive to another sural nerve graft procedure.

### Disability of the reconstructed nerve injury and its impact on leisure, school and work activities

The patients considered the overall disability (VAS 0–100) from their reconstructed nerve injury as low [25 (0–100)], with little impact on their leisure activity, school or work activities [8.5 (0–100) and 11.5 (0–100), respectively].

### Residual symptoms and correlation with general outcome of nerve reconstruction and age

The results of the scoring system of residual symptoms in the leg showed a low value [3.5 (0–13)]. The residual symptom score did correlate with the VAS score of the general outcome (rho-value = 0.45; p = 0.004), but did not correlate with the time of follow up (p = 0.39). Age at injury did not correlate with general outcome or residual symptoms scores (p > 0.05). In addition, there were no differences in residual symptoms (p = 0.86) or general outcome of reconstruction of the nerve injury (p = 0.43) between subjects that had their injured nerve reconstructed with a sural nerve graft at an age ≤ or > 20 years of age.

## Discussion

The present study shows that symptoms in the lower leg after harvesting the sural nerve for reconstructive surgery are relatively modest and include sensory loss, tingling sensations and sensitive to cold. More than half of the patients did not experience any discomfort immediately following surgery and none of the patients in this study had any postoperative complications from harvesting the sural nerve. With this in mind, the nerve harvest procedure can be considered a safe procedure in healthy individuals. The results correlate well with previously published studies [5-9,11], where it was concluded that the majority of patients tolerate harvest of a sural nerve well and that there are only minor persisting symptoms from the donor site, which was irrespective of the length of follow up. Such statement has also been emphasized in paediatric patients [7].

The subjectively most common symptom, as expected [5] and experienced by over 90% of the present patients, was some degree of sensory loss at the foot most commonly in the skin around the heel (Figure 1), which correspond well with our previous results [4,7-10], where the same questionnaire was used to evaluate sequele after biopsy of the whole sural nerve. Interestingly, the patients experienced that the area of sensory loss in the skin decreased over time. In accordance, healthy subjects also notice a similar phenomenon after a sural nerve biopsy [4,9,10,12]. Previous studies, based on telephone interviews, did not focus on reduction of the sensory loss [6,11]. The gradual decrease of the area of sensory loss may depend on a combination of collateral sprouting from sensory nerves adjacent to the cutaneous area formerly innervated by the sural nerve [9,12] and brain plasticity [6,9,13]. Information, regarding the possibility for sensory loss around the heel and lateral foot and that this area may decrease over time as well as information about mild or intermittent allodynia seen in 50% of the patients, should be provided to the patients before harvesting the sural nerve. The experienced pain, both immediately and years after the sural nerve harvesting, was described mostly as mild or none at all, which is consistent with our previous study on symptoms following a sural nerve biopsy in healthy volunteers [10]. Interestingly, there was no need among our patients to presently use potent painkillers. In few patients (n = 3) the sural nerve was harvested bilateral. Thereby, it was not possible to statistically test for any differences in subjective outcome of the harvest procedure or to ask them to compare with the contralateral side. In addition, we anticipated that the patients operated on bilaterally responded on the subjective outcome of the procedure as related to their possible worse side. This is a limitation and in future use of this questionnaire it should be clearly stated.

The overall score, how the patients experienced their general outcome of the reconstructed nerve [median = 25; highest score 100], were rather low (i.e. limited problems) and with minor problems from the donor site [median = 3; maximal score 16]; scores that correlated with each other, i.e. a worse result of the reconstruction procedure correlated positively with residual problems from the sural nerve harvest. Such a finding may be understandable, but one should also consider psychological mechanisms in this context; thus, a poor result may lead to that the patient experience more problems from the leg. A number of patients (37%) had demanding physical job, such as factory worker or auto mechanic, but the impact of the nerve injury on their work capability was surprisingly low. Two patients were unable to return to their regular work. Their work tasks included lifting of heavy objects and requirements to work manually with equal use of both arms and hands. However, the main reason that they had to change occupation was the severe nerve injury in their upper extremities requiring nerve grafting. Miloro et al. [5] described an association between an age >38 years and a worst general outcome locally in the leg after harvesting the sural nerve, but we did not see any difference in residual symptom or general outcome score in the patients with an age ≤ or > 20 years of age (i.e. injury during childhood and adolescence) at the time of reconstruction. In addition, age did not correlate with residual symptoms or general outcome score. Thus, such information should be provided to the patients that undergo a nerve reconstruction procedure. Harvest of the sural nerve for nerve reconstruction is a safe procedure also in paediatric patients [7]. Recently, it was reported that outcome of nerve repair in the forearm is worse when such a nerve injury is repaired or reconstructed after the age of 12 years [14]. Notably, most of the present patients were above 12 years of age, which may influence the results.

There are also other limitations of the study since some patients were operated on 30 years ago [8]. They may have difficulties to remember the immediate postoperative symptoms. Interestingly, the length of follow up did not correlate with the residual symptoms (i.e. value of total score). Another limitation is that the patients included had various nerve injuries, extending from a pure nerve injury to a complete amputation of their forearm or even a brachial plexus injury. Such factors may influence how the patients experience their illness and thus their answers in the questionnaire. However, according to the response, the experience of residual symptoms in the foot or leg after harvesting the sural nerve is not dependent on type of nerve injury. Notably, here we used a questionnaire sent to the patients allowing them to unbiased fill out the questions. A previous study [6] has been based on answers from a telephone questionnaire and there is a possibility that the subjects may be influenced by the person interviewing them.

As always, it is a matter of balancing benefits and risks against each other in nerve reconstruction procedures. In the case of a new nerve injury, 36 out of 41 patients would accept an additional sural nerve grafting procedure if necessary. The positive attitude to further surgery can be explained by that the majority of the patients´ injuries in their extremities affect their daily living much more than the residual symptoms that arise after harvesting the sural nerve. In contrast, a reduced willingness to perform an additional sural biopsy from the contralateral side is low among subjects with or without diabetes although they may have similar symptoms after the biopsy [4,10] (Table 2). Only 5% graded their symptoms as severe, but none of the patients in any of the studies experienced symptoms disturbing sleep [4,10]. Thus, patients, who had a whole sural biopsy, were not nearly as willing to undergo a further harvest of the sural nerve [7/21 (33%)] as the patients in the present study [36/41 (88%)], which is reasonable since the present patients would have a greater gain from their nerve graft reconstruction.

## Conclusions

We conclude that harvest of a sural nerve to reconstruct an injured nerve trunk is a safe procedure with mild residual symptoms similar to those seen after a sural nerve biopsy, but such patients are less prone to undergo an additional biopsy.

## Abbreviations

VAS: Visual analogue scale.

## Competing interests

The authors have no competing interests related to the present study, except that LD has received reimbursements from AxoGen Inc, FL, USA.

## Authors’ contributions

LD and ABN designed the study. AH, together with ACZ, collected all questionnaires. AH assembled the results and wrote the manuscript together with the other authors. All authors approved the final submitted manuscript.

## Pre-publication history

The pre-publication history for this paper can be accessed here:

http://www.biomedcentral.com/1471-2482/13/39/prepub

## Supplementary Material

Additional file 1 Questionnaire. Click here for file
