# Supplementary material for: Subjective outcome related to donor site morbidity after sural nerve graft harvesting: a survey in 41 patients
Source: BMC Surg. 2013 Sep 24;13:39. doi: 10.1186/1471-2482-13-39 (PMC3848941; doi:10.1186/1471-2482-13-39)
Supplement: Additional file 1 — Questionnaire. [file 1471-2482-13-39-S1.pdf]

Questionnaire – follow up after harvesting the sural nerve for nerve reconstruction

1. Did you have any discomfort in the foot directly after the operation?

Yes

No

No reply

2. Did you experience any loss of sensation in the operated area after the operation?

Yes

No

3. Did you experience pain in the operated area after the operation?

Yes

No

No reply

4. Do you have loss of sensation in the operated foot compared with the other foot?

Yes

No

No reply

5. Mark the area of sensory deficit in the figure.

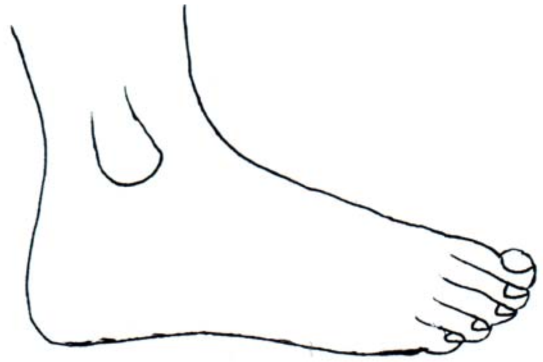

6. (a) Has the area with loss of sensation decreased compared with the time directly following surgery?

Yes

No

No reply

(b) If yes, how much (%)

0-25

26-50

51-75

76-100

7. (a) Do you feel pain in the foot/lower leg?

Yes

No

No reply

(b) When?

Day time

Night time

Day time and Night time

8. (a) Do you have problems with cold intolerance in the operated foot/lower leg?

Yes

No

No reply

(b) If yes, how often?

Frequently

Sometimes

Rarely

9. (a) Have you experienced problems with increased skin sensation when the skin is touched?

Yes

No

(b) If yes, how often?

Frequently

Sometimes

Rarely

10.(a) Do you experience discomfort or

tingling along the outside of the foot?

Yes

No

(b) If so, when do these symptoms occur?

At rest

During walking

Impact against surgical site

11. How would you describe your

problems at the moment?

Disturbed sleep

Powerful

Affecting daily living

Mild

None

12. Do you have to take painkillers often?

Yes

No

13. (a) Do you have any disease that can

affect the nervous system, for example;

diabetes, vitamin deficiency or thyroid

disease.

Yes

No

(b) If yes, which?

14. A theoretical question: would you be positive to have your other sural nerve harvested if you had to undergo another nerve reconstructive surgery?

Yes

No
